# Good functional outcomes after endoscopic treatment for greater trochanteric pain syndrome

**DOI:** 10.1186/s40634-023-00574-3

**Published:** 2023-03-15

**Authors:** Louise Karlsson, Philip Quist, Katarina Nilsson Helander, Thorkell Snaebjörnsson, Anders Stålman, Ida Lindman, Axel Öhlin

**Affiliations:** 1 Department of Orthopedics, Sahlgrenska University Hospital Mölndal, Institute of Clinical Sciences, Sahlgrenska Academy, Gothenburg, Sweden; 2 School of Medical Sciences, Örebro University, Örebro, Sweden; 3 Capio Artro Clinic, FIFA Medical Centre of Excellence, Stockholm Sports Trauma Research Center, MMK, Karolinska Institutet, Stockholm, Sweden; 4 Department of Molecular Medicine and Surgery, Stockholm Sports Trauma Research Center, Karolinska Institutet, Stockholm, Sweden

**Keywords:** GTPS, Trochanteric bursectomy, Arthroscopy, Fascia latae plasty, Patient reported outcome measures, iHOT-12

## Abstract

**Purpose:**

Greater trochanteric pain syndrome (GTPS) is a term covering different conditions generating lateral hip pain. Recalcitrant cases may require surgery but there are only a few studies evaluating endoscopic treatment. This study aimed to evaluate the outcome of endoscopically treated GTPS at minimum two years postoperatively using patient-reported outcome measures (PROMs), and to assess the complication rate associated with endoscopic surgery.

**Methods:**

A total of 33 patients, mean age 43.2 years, 88% women, with a mean symptom duration of 3.5 years, were included in the study. A total of 36 operated hips were included. Pre- and at minimum two years postoperatively the patients completed questionnaires consisting of the International Hip Outcome Tool (iHOT-12) and the Hip Sports Activity scale (HSAS), the Visual analogue scale for overall hip function (VAS-OHF), the Copenhagen Hip and Groin Outcome Score (HAGOS), the EuroQoL-5 Dimension Questionnaire (EQ-5D) and the EQ-VAS. Complications were assessed using the Clavien-Dindo classification.

**Results:**

Median follow-up time was 24.5 months postoperatively. Statistically significant improvements were seen for the following PROMs (*p* < 0.05); iHOT-12 (36.3 vs 54.0), HAGOS different subscores (40.8 vs 59.0, 46.5 vs 62.6, 29.9 vs 53.1, 33.5 vs 51.4, 20.7 vs 41.4, 23.4 vs 43.3), EQ-VAS (55.9 vs 63.3) and EQ-5D (0.392 vs 0.648). VAS-OHF and HSAS did not reach significance. There was a 71% satisfaction rate with the surgery. Three Clavien-Dindo grade 1 and one grade 2 complications were registered postoperatively, with 41% of patients achieving PASS for iHOT-12 at two years follow-up.

**Conclusion:**

Endoscopic surgery for greater trochanteric pain syndrome improved patient-reported outcomes and the procedure was associated with low risk of complications.

**Level of evidence:**

Level IV.

## Background

Historically, chronic peritrochanteric pain radiating along the lateral border of the hip, with tenderness to palpation, has been termed trochanteric bursitis [28]. Currently, the term Greater trochanteric pain syndrome (GTPS) is instead adopted in the literature. An umbrella term, GTPS covers gluteus medius/minimus tendinopathy, iliotibial band (ITB) disorders, such as external coxa saltans, and trochanteric bursitis [2]. The incidence of GTPS ranges between 1.8 and 5.6 per 1000 inhabitants per year in the US. Affected patients, are most often women of menopausal age, with a ratio of 4:1 as regards women to men [15, 24].

The primary treatment for GTPS is mainly non-surgical. When non-operative treatments have failed, surgical management could be considered [9, 10, 17, 24, 27]. Endoscopic surgery for GTPS with trochanteric bursectomy was first described in 1998 [3]. Subsequent studies have described positive outcomes, with reports of reduced pain and improved hip function after Endoscopic treatment for GTPS [5, 11, 27]. However, previous studies have been small, and used different surgical techniques as well as patient reported outcome measures (PROMs) not suitable for patients undergoing hip arthroscopy, such as the modified Harris Hip Score (mHHS) [6–8, 17, 26, 29]. The Copenhagen Hip and Groin Outcome Score (HAGOS) and the international Hip Outcome Tool (iHOT-12 or iHOT-33, containing either 12 or 33 questions) are PROMs developed to accurately mirror the hip function in young and active patients with hip problems and are validated and translated to Swedish [12, 25]. In 2011, a hip arthroscopy registry was initiated in Gothenburg, Sweden, using a set of PROMs to evaluate treatment outcome. The PROMs consist of the iHOT-12, VAS for overall hip function (VAS-OHF), HAGOS, European Quality of Life – 5 Dimension Questionnaire (EQ-5D), EQ-VAS, the hip sports activity scale (HSAS) and a single question on the patients’ satisfaction with their surgery [22].

The primary aim of the present study was to evaluate the patient reported outcomes at minimum two years postoperatively in a cohort of patients treated for recalcitrant GTPS with Endoscopic surgery, with iHOT-12 used as the primary outcome measurement.

A secondary aim was to examine the occurrence of complications associated with the surgery.

## Methods

### Patients and methods

**Fig. 1:**
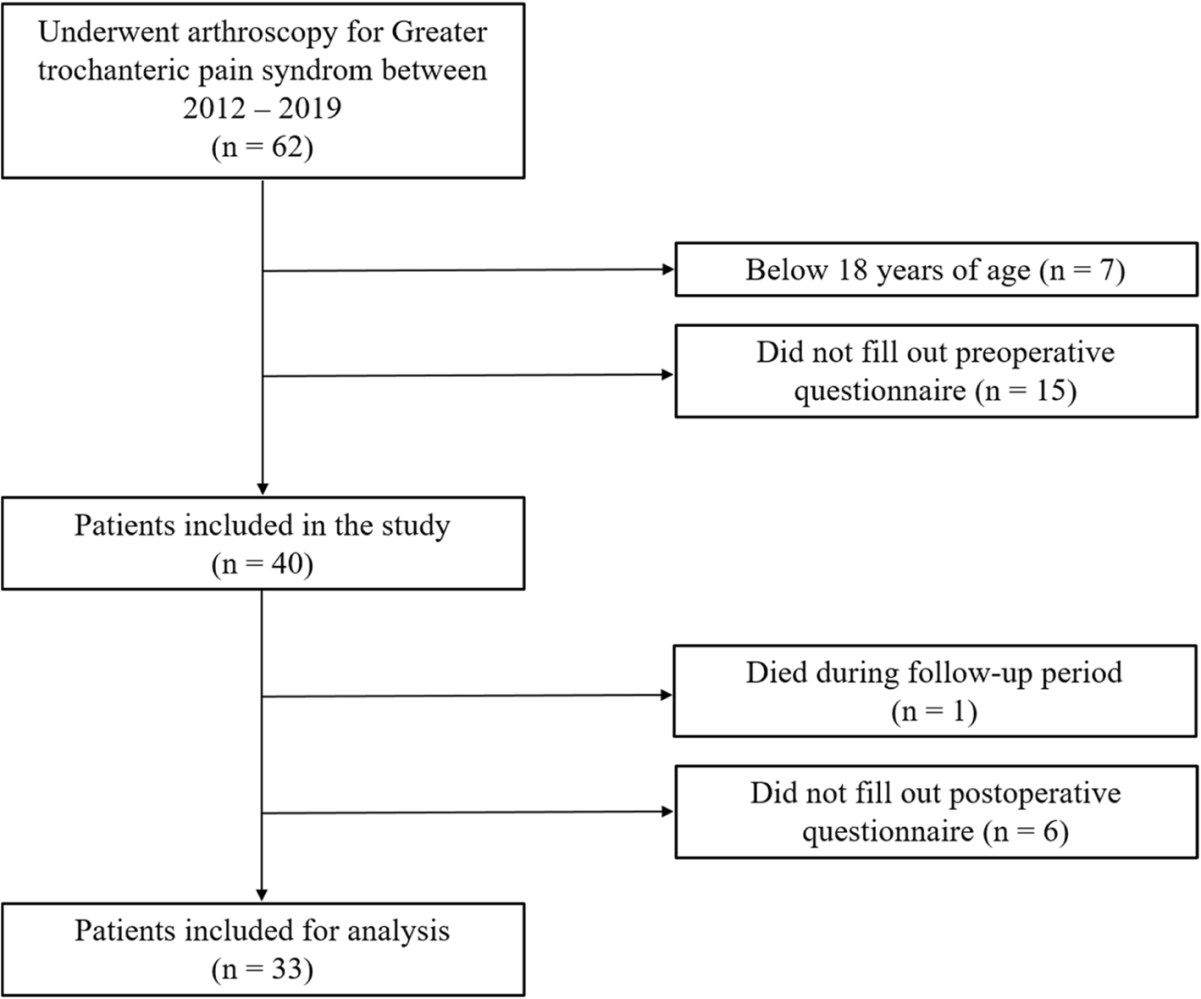
Flow chart describing patient inclusion and follow-up

Retrospective analysis of prospectively collected data from the Gothenburg hip arthroscopy registry for the years 2012 to 2019 was used in this study. There were 62 patients identified in the registry that underwent endoscopic surgery with trochanteric bursectomy and/or fascia latae plasty for the diagnosis GTPS. These patients were all eligible for inclusion in this study. An established diagnosis of recalcitrant GTPS with unsuccessful non-surgical treatment, consisting of physiotherapy and with or without corticosteroid injections, was the indication for surgery. The diagnosis was made from physical examination and patient history. Surgeries were performed by five surgeons at three hospitals in Gothenburg, Sweden. The exclusion criteria were age under 18 at time of surgery and/or no preoperative or postoperative PROMs. Please see Fig. 1 for flow chart of inclusion and exclusion.

The iHOT-12 was used as the primary outcome. Patients that had not responded to the follow up at two years were contacted by phone during the fall of 2021 for the purpose of this study, and asked to fill out the questionnaire anew.

Supplementary patient data, including the number of re-operations and complications, were retrieved from patient journals. The Clavien-Dindo classification was used to assess complications associated with the operation [4]. The Clavien-Dindo classification is a common way to describe surgical complications. It is a five-leveled scale ranging from 1 (deviation from normal postoperative course without need for certain pharmacological or surgical treatment) to 5 (death). Grade 2 complications include surgical wound site infections requiring antibiotics, grade 3 includes complications that require surgical intervention and grade 4 includes complications requiring intensive care.

### Surgical technique – trochanteric bursectomy and fascia latae plasty

With the patient supine two portals were routinely used, placed at the level of the long axis of the femur. They were about the same distance from the most lateral aspect of the trochanter, one caudal and one distal. Subsequently, the troachar was introduced subcutaneously through the proximal portal and advanced to the fascia latae, which was bluntly freed from the subcutaneous tissue. The arthroscope was then introduced, and the water turned on, to open a space directly outside the fascia latae. The other distal portal was used for the shaver and electric cautery device. Next, the most lateral point of the trochanter was identified with use of a c-arm and the fascia latae was longitudinally excised above and below the most lateral extent of the trochanter to a total of 7–10 cm. Further, the trochanteric bursa, lying directly behind the fascia latae, was resected with a shaver and bleeding vessels were cauterized. The trochanter could then be visualized and inspected for gluteus medius tears. If signs of gluteus medius damage or insufficiency were found, no fascia latae lengthening was performed to avoid risk of abduction weakness, and these patients only underwent trochanteric bursectomy. If there were no signs of gluteus medius damage, a second incision was made in the fascia latae at 90 degrees from the first one. This incision was centered on the trochanter, spanning about 1–2 cm ventrally and 2–3 cm posteriorly to form a plus ( +) sign in order to achieve fascia latae lengthening.

Postoperatively, full ROM and weight bearing was allowed, and postoperative rehab focused on regaining normal walking pattern as soon as possible, as well as hip and core strength and stability.

### Statistical analysis

Patient demographics were analyzed with descriptive statistics and expressed as median, range, mean and standard deviation (SD). Categorical variables were expressed in relative and absolute frequencies. Non-parametrical testing was applied to evaluate PROM data, as data was not normally distributed. Preoperative values and data obtained at follow-up were thus compared with the Wilcoxon signed rank test [23].

To evaluate clinically relevant changes, minimal important change (MIC) was adopted for HAGOS and iHOT-12. The definition of MIC is the minimal change in outcome scores that the patient would appraise as important, and was calculated as 0.5 × SD of the mean of change [14, 18, 21]. The patient acceptable symptomatic state (PASS) was set to an iHOT-12 value of 63 at the two-year follow-up [19].

Statistical analyses were performed with SPSS (IBM Corp. Released 2019. IBM SPSS Statistics for Macintosh, Version 26.0. Armonk, NY: IBM Corp.). Level of significance was set at *p* < 0.05 and all significance tests were two-tailed.

## Results

Out of 62 patients treated for GTPS between 2012 and 2019, 33 patients undergoing 36 surgical procedures were included for analysis in this study. See Fig. 1 for a flow chart of included patients. Two patients underwent simultaneous bilateral hip arthroscopies and one patient were treated bilaterally, but with two years interval, generating a total of 34 PROM values in the analysis. See Table 1 for demographics. The mean (SD) age overall was 43.2 (± 15.5) years, with a range of 21 – 72 years. Of the 36 hips surgically treated for GTPS, 23 (64%) hips were treated for isolated GTPS and 13 (36%) hips for GTPS with simultaneous FAIS. All patients but the 5 that had gluteus medius rupture underwent fascia latae plasty along with their trochanteric bursectomy. See Table 2 for surgical procedures.

**Table 1:** Patient demographics

Demographics	
Number of patients	33
Number of hips	36
Operated side, L/R/Bilateral (%)	12/18/3 (36/55/9)
Female/male (%)	29/4 (88/12)
Age – mean (SD)	43.2 (15.5) years
Symptom duration^a^ – median (min–max)	3.5 (1 – 21) years
Follow-up time – median (min–max)	24.5 (24 – 100) months
Previous ipsilateral hip surgery, %	
Trochanteric bursectomy and/or fascia latae plasty	3
FAIS surgery	5
Trochanteric bursectomy and/or fascia latae plasty + FAIS surgery	1
Gluteus medius and/or minimus tendon injury^b^ Degenerative/Rupture (%)	3/2 (8/6)

*L* Left, *R* Right, *SD* Standard deviation, *FAIS* Femoroacetabular impingement syndrome

^a^Data available on 30 patients

^b^Registered during surgery

**Table 2:** Endoscopic procedures performed

Procedure	Hips (%)
**Trochanteric bursectomy + Fascia latae plasty**	
** Isolated**	23 (64)
** + Cam resection**	6 (17)
** + Pincer resection + labrum resection**	1 (3)
** + Cam and Pincer resection**	5 (14)
** + Cam and Pincer resection + labrum suture**	1 (3)

Prior to their GTPS surgery, 9 out of 36 hips (25%) had undergone previous hip surgery. Of these, four patients had undergone previous trochanteric bursectomy and/or fascia latae plasty, three open surgeries between seven and 20 years prior to the endoscopic treatment in this study. The fourth patient had undergone open trochanteric bursectomy 20 years prior to their endoscopic treatment, as well as arthroscopic cam resection in the same year as the endoscopic treatment for GTPS. The other patients had undergone hip arthroscopy for FAIS, where three arthroscopies were performed seven years earlier or more, four approximately two years prior to the studied surgery and two one year before the studied surgery. Further baseline characteristics are displayed in Table 1.

**Fig. 2:**
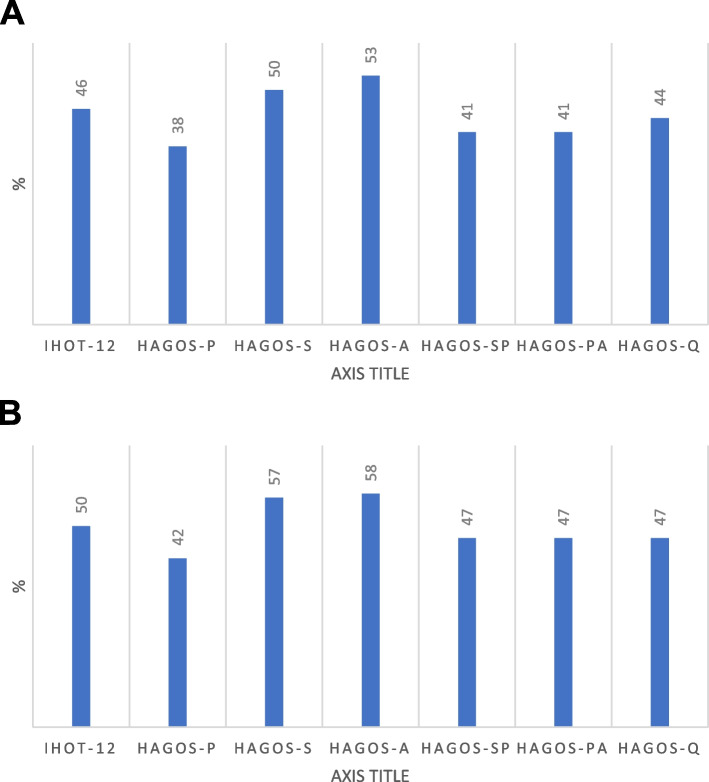
**a** Distributions of patients in the total study population exceeding the minimal important change (MIC) value. *iHOT-12* International hip outcome tool, *HAGOS* Copenhagen hip and groin outcome score, *P* Pain,* S* Symptoms, *A* Daily activity, *SP* Sports, *PA* Physical activity, *Q* Quality of life. b Distributions of patients operated with trochanteric bursectomy and fascia latae plasty, exclusively, exceeding the minimal important change (MIC) value. *iHOT-12* International hip outcome tool, *HAGOS* Copenhagen hip and groin outcome score, *Q* Quality of life, *S* Symptoms, *P* Pain, *PA* Physical activity, *SP* Sports, *A* Daily activity

In total, when comparing outcomes pre- and postoperatively, statistically significant improvements were obtained for all PROMs except VAS-OHF and HSAS (Table 3). The proportions of patients exceeding the MIC for iHOT-12 and HAGOS are demonstrated in Fig. 2a. A total of 71% of responding participants (3 missing values) reported satisfaction with the surgery. Of the 28 patients that responded to iHOT-12 at the two-year follow-up, 11 patients surpassed the PASS set at 63, which amounts to 41% of the responding cohort achieving an acceptable symptom state.

**Table 3:** Outcome scores, all patients

Outcome	Preoperative Mean (SD)	Follow-up Mean (SD)	Change (SD)	*p* value	MIC
iHOT-12^#^	36.3 (14.5)	54.0 (31.6)	17.7 (25.7)	**0.007**	12.9
VAS – overall hip function*	49.1 (20.9)	58.5 (32.5)	9.39 (35.0)	0.141	N/A
EQ-VAS^+^	55.9 (17.3)	63.3 (20.7)	7.31 (20.4)	**0.034**	N/A
EQ-5D^+^	0.392 (0.325)	0.648 (0.310)	0.257 (0.368)	**0.002**	N/A
HAGOS – pain^+^	40.8 (17.9)	59.0 (25.9)	18.2 (26.9)	**0.001**	13.5
HAGOS – symptoms^+^	46.5 (17.6)	62.6 (27.1)	16.1 (25.2)	**0.002**	12.6
HAGOS – daily activity^+^	29.9 (28.0)	53.1 (37.4)	23.2 (33.2)	**0.001**	16.6
HAGOS – sport^+^	33.5 (23.9)	51.4 (31.2)	17.9 (27.6)	**0.003**	13.8
HAGOS – physical activity^+^	20.7 (18.7)	41.4 (35.3)	20.7 (34.0)	**0.004**	17.0
HAGOS – quality of life^+^	23.4 (13.8)	43.3 (30.8)	19.8 (28.7)	**0.002**	14.4
HSAS*	1.74 (1.71)	2.26 (1.48)	0.516 (1.90)	0.061	N/A

Bold numbers indicate statistically significance

*SD* Standard deviation, *MIC* Minimal Important Change, *N/A* Not applicable, *iHOT-12* International Hip Outcome Tool, *VAS V*isual Analogue Scale, *EQ-5D* EuroQoL-5 Dimension Questionnaire, *HAGOS* Copenhagen Hip and Groin Outcome Score, *HSAS* Hip Sports Activity Scale

^#^6 missing values

^*^3 missing values

^+^2 missing values

When analyzing the isolated trochanteric bursectomy and fascia latae plasty procedures exclusively, improved outcome scores with statistically significant changes were seen post-surgery for all PROMs, except HSAS (Table 4). Distribution of patients treated for isolated GTPS that exceeded the MIC for iHOT-12 and HAGOS are shown in Fig. 2b. The level of satisfaction with the surgery in this subgroup was 78%.

**Table 4:** Outcome scores, isolated trochanteric bursectomy and fascia latae plasty

Outcome	Preoperative Mean (SD)	Follow-up Mean (SD)	Change (SD)	*p* value	MIC
iHOT-12^#^	36.0 (15.1)	54.9 (30.0)	18.9 (22.3)	**0.006**	11.2
VAS – overall hip function*	47.9 (22.1)	67.8 (22.7)	19.9 (27.2)	**0.012**	NA
EQ-VAS^+^	56.1 (18.4)	65.3 (19.1)	9.21 (13.5)	**0.012**	NA
EQ-5D^+^	0.435 (0.322)	0.687 (0.264)	0.252 (0.327)	**0.007**	NA
HAGOS – pain^+^	41.8 (18.5)	59.6 (24.4)	17.8 (21.8)	**0.003**	10.9
HAGOS – symptoms	47.6 (19.2)	64.5 (26.4)	16.8 (23.6)	**0.006**	11.8
HAGOS – daily activity^+^	32.2 (29.6)	54.3 (35.6)	22.0 (27.7)	**0.003**	13.9
HAGOS – sport^+^	35.9 (27.6)	53.9 (30.8)	18.1 (22.3)	**0.003**	11.2
HAGOS – physical activity^+^	21.1 (21.3)	42.8 (32.4)	21.7 (33.8)	**0.019**	16.9
HAGOS – quality of life^+^	24.5 (13.8)	45.5 (27.2)	21.1 (25.6)	**0.003**	12.8
HSAS*	1.72 (1.49)	2.39 (1.54)	0.667 (1.88)	0.105	NA

Bold numbers indicate statistically significance

*SD* Standard deviation, *MIC* Minimal Important Change, *N/A* Not applicable, *iHOT-12* International Hip Outcome Tool, *VAS V*isual Analogue Scale, *EQ-5D* EuroQoL-5 Dimension Questionnaire, *HAGOS* Copenhagen Hip and Groin Outcome Score, *HSAS* Hip Sports Activity Scale

^#^5 missing values

^*^3 missing values

^+^2 missing values

During the follow-up period, one re-operation was performed due to inadequate improvement. Three Clavien-Dindo classification grade 1 and one grade 2 complications were registered. The reoperation was not considered a complication in the aforementioned classification. The complications consisted of fluid accumulation causing unusual amount of pain and bleeding from the surgical wound site (grade 1) and a superficial wound infection demanding antibiotics (grade 2).

## Discussion

The main findings in this study were that patients displayed significant improvements at minimum two-years follow-up after endoscopic treatment for GTPS regarding the primary outcome iHOT-12, and that 71% of the patients were satisfied with the surgery. Another key finding of the present study was that endoscopic surgery for GTPS was associated with a low risk of complications.

A limited number of studies have evaluated the short- to mid-term outcomes following arthroscopic surgery for GTPS and the majority are small retrospective studies. These studies have reported predominantly favorable results. In a prospective study, Baker et al. evaluated the outcome of endoscopic trochanteric bursectomy and concomitant ITB-release [1]. They reported, in a cohort of 25 patients with a mean follow-up time of 26 months, comparable results to the present study, a reduced VAS value, and an improved patient-reported hip function measured with Harris Hips Score (HHS).

The Harris Hip Score (whether modified or not), however, has been shown to be less suited for use in patients undergoing arthroscopic hip treatment, especially young and active, as it was developed for use in mostly elderly patients undergoing total hip arthroplasty [13]. Further, the opportunity to compare the present study to previous results is limited due to the use of different PROMs.

Several recent papers on the outcome of trochanteric bursectomy and fascia latae plasty have implemented gluteal tendon repair when discovering an injury to the gluteus medius tendon, which was not performed in the present study [5, 6, 26, 12]. In the study published in 2016 by Drummond et al., it was reported on statistically significant improvements in pain and hip functions on 49 patients who had undergone endoscopic ITB release, trochanteric bursectomy and, in 7 cases, gluteal tendon repair together with platelet rich plasma tendon injections [9]. Overall mean VAS decreased while Oxford hip Score and iHOT-33 increased. The improvements of the iHOT-33 score corresponds to the mean change of iHOT-12 seen in this study. Further, the reported distribution of patients satisfied with the surgery was 79%, which is comparable with the present study.

In the present study, there was a low number of patients experiencing postoperative complications. A systematic review published in 2016 reported unanimously outcomes with no major complications associated to endoscopic trochanteric bursectomy and low numbers for ITB-release [21].

The HSAS level was left with no significant change post-surgery. This could be due to a floor effect since this patient group had a low preoperative physical activity level.

The main strengths of the present study are the mid-term follow-up period and the prospectively collected data. This study also describes one of the largest patient populations undergoing endoscopic treatment for GTPS. The use of multiple validated outcome scores, constitutes an additional strength.

An aspect lacking in many previous studies, was that a minority of the patients included were treated endoscopically for GTPS and FAIS simultaneously. This can make it difficult to discriminate which of the two procedures was of greatest benefit for the patient. However, similar tendencies in mean change were seen when comparing the total population and isolated GTPS procedures. Nevertheless, GTPS has been called “the great mimicker” and to treat the diagnosis on the right indications can be a challenge to clinicians [20]. In some cases, GTPS and FAIS may occur simultaneously and in some patients with suspected FAIS, GTPS may be a hidden cause of hip pain, particularly under the age of 40 [20]. Thus, involving patients operated with both GTPS and FAIS procedures may reflect the clinical reality more accurately, and can be argued to be a strength to the present study design.

There are some limitations to this study. The small sample size carries a risk of type II errors, especially in the isolated GTPS subgroup. Of the 62 surgical procedures, only 36 could be included in the final analysis as the other patients either had not filled out PROMs pre- or postoperatively, or were under the age of 18. This large loss to follow-up can be seen as a complication. However, a previous dropout analysis has been done on the registry where the registry was deemed to have good external validity [16]. No power calculation was conducted beforehand, with all patients treated with trochanteric bursectomy in the local hip arthroscopy registry being eligible for inclusion. Secondly, in spite of long symptom duration which limits the return to mean effect, it is impossible to ignore the risk of a placebo or another independent effect on the results, due to the lack of a control group. A few patients had also undergone previous trochanteric bursectomy and/or fascia latae plasty (all open) before inclusion in this study. This could be seen as a limitation as their previous surgery could bias their result from the one performed in the present study. It is also unknown in the scope of the registry whether all patients became symptom free after their first surgery or if they had persistent symptoms. Since more than seven years had passed for every patient between their surgeries however, they were chosen to be included in this study. The reason in the present study to include all patients undergoing endoscopic surgery for GTPS was to better mirror the surgical reality, which raises the external validity of this study.

Moreover, the number of complications and re-operations were only obtained from the journals at the clinic where the index surgery was performed. As a result, complications and re-operations discovered and performed at other clinics may have been missed. The patients were, however, provided with a thorough follow-up and therefore the risk of having events not detected during the follow-up period is minimal.

To evaluate clinically relevant improvements, the MIC was calculated using the standard deviation of the mean change, however, since these figures were skewed, the proportion of patients exceeding MIC in this present study might be more difficult to interpret. To prevent this, an anchor-based calculation model could have been used instead, which was not possible in the present study as there was no anchor included in the PROMs to be used as a reference. To evaluate PASS, a previous study calculated the iHOT-12 value at 63 at one-year follow-up for patients undergoing hip arthroscopy for FAIS. PASS for iHOT-12 has not been validated for GTPS however, which could make it difficult to determine the clinical relevance of the results in the present study. Interestingly, 71% of the present cohort felt satisfied with the surgery, which could be a more clinically relevant result as the question directly associates to the treatment for GTPS.

## Conclusion

In conclusion, a statistically significant improvement in hip function, pain and quality of life was seen at a minimum of two years following endoscopic treatment for GTPS. The majority of the patients (71%) were satisfied with the surgery and the procedure was associated with low risk for major complications.

## Data Availability

The datasets generated and analyzed during the current study are not publicly available due to containing individual social security numbers on included patients, but can be available from the corresponding author on reasonable request.
